# Histological observation of goblet cells following topical rebamipide treatment of the human ocular surface: A case report

**DOI:** 10.3892/etm.2014.2108

**Published:** 2014-12-05

**Authors:** SATORU KASE, TOSHIYA SHINOHARA, MANABU KASE

**Affiliations:** 1 Department of Ophthalmology, Teine Keijinkai Hospital, Sapporo 006-0811, Japan; 2 Department of Surgical Pathology, Teine Keijinkai Hospital, Sapporo 006-0811, Japan

**Keywords:** rebamipide, goblet cells, mucin-like substance, humans, histopathology

## Abstract

The topical administration of rebamipide (Mucosta®), an antiulcer agent, clinically increases the mucin level of tear film. The aim of this study was to report the histological changes of goblet cells following the topical administration of rebamipide to a patient with nevus of the lacrimal caruncle. A 62-year-old male exhibited a pigmented nodule located in the lacrimal caruncle in the left eye. An excisional biopsy and subsequent surgical resection were conducted at the caruncle, prior to and three months after topical rebamipide administration. Histologically, a biopsy specimen revealed a pigmented nevus beneath the caruncle epithelium containing a few goblet cells [4 cells/high power field (HPF)]. A few nevus cells were present at the surgical margin. By contrast, the secondary resected specimen obtained three months after the initiation of topical rebamipide treatment revealed the epithelium and nevus, where numerous goblet cells were present (28 cells/HPF), and mucin-like substances were markedly secreted from the goblet cells. Topical rebamipide markedly increased the number of goblet cells and stimulated the secretion of mucin-like substances in the caruncular tissue of a human patient. These results suggest that topical rebamipide is useful in patients following surgery and/or biopsy to support tissue repair of the ocular surface.

## Introduction

Goblet cells synthesize, store and secrete a complex of high-molecular-weight glycoproteins. Mucins, included in the glycoproteins, are essential for the maintenance of a normal precorneal tear film and healthy ocular surface (1). It has been demonstrated that the topical administration of rebamipide (Mucosta^®^), an antiulcer agent, increases the mucin level of tear film, and exclusively contributes to improvement of the ocular surface in patients with dry eye (2). A study has shown that rebamipide increases the number of goblet cells in the bulbar conjunctiva of the rabbit *in vivo* (3). Rios *et al* demonstrated that rebamipide led to the proliferation of cultured rat conjunctival goblet cells (4), subsequently stimulating secretion from the cells (5). Topical rebamipide has been shown to significantly ameliorate corneal epithelial damage, by enhancing the expression of mucin *MUC5AC* mRNA on the murine ocular surface (6). Clinically, the topical administration of rebamipide increases the mucin level of tear film, and improves the ocular surface in dry eye (2). We recently revealed markedly increased goblet cell numbers following the administration of topical rebamipide in a patient with conjunctival dysplasia (7). However, the mechanism by which rebamipide exerts a strong action on goblet cell behaviors and mucin secretion in human ocular surface remains largely unknown how. To the best of our knowledge, the present study describes the first case of nevus in the lacrimal caruncle showing a marked increase in goblet cell number and secretion of mucin-like substances following the administration of topical rebamipide.

## Case report

**Figure 1:**
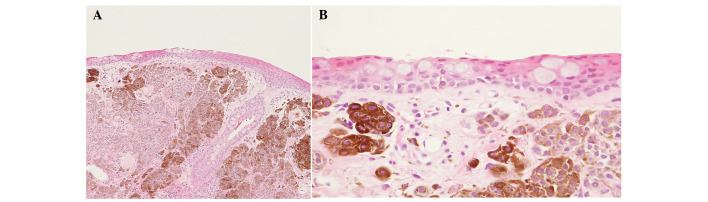
Histological findings of the lacrimal caruncle prior to topical rebamipide administration. (A) There are pigmented nevus cells beneath the epithelium (magnification ×100). (B) Several goblet cells are noted within the epithelium (arrows). Mucin-like substances are not observed upon the epithelium (magnification, ×400).

**Figure 2:**
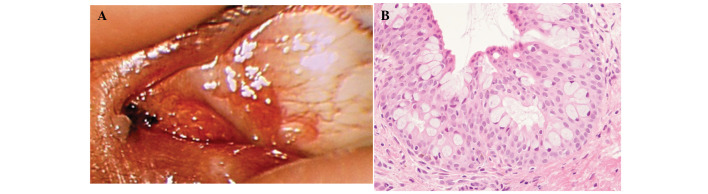
Histological and slip-lamp examination findings of the lacrimal caruncle following rebamipide administration. (A) A slit-lamp examination reveals a tiny pigmented lesion in the lacrimal caruncle. (B) At high magnification of the biopsy specimen, the noncancerous epithelium contains numerous goblet cells (28 cells/high power field), where marked secretion of mucin-like substances from the goblet cells is noted (arrows). Magnification ×400.

A 62-year-old male complained of enlargement of a pigmented lesion in the left eye. The patient was referred to Teine Keijinkai Hospital (Sapporo, Japan) due to the observation of a lacrimal caruncle tumor at a local clinic. The visual acuity of the patient was 1.0 for the left eye with normal intraocular pressure. Slit-lamp examination revealed a pigmented tumor located in the caruncle. The right eye was normal. Since nevus or malignant melanoma was initially suspected, an excisional biopsy was conducted on July 11, 2013. Histologically, since there were pigmented nevus cells beneath the epithelium without cellular atypia (Fig. 1A), the tumor was diagnosed as pigmented nevus. A few goblet cells were noted within the epithelium [4 cells/high power field (HPF); Fig 1B, arrows]. Mucin-like substances were not observed on the epithelium (Fig. 1B). Nevus cells were present at the surgical margin. The patient underwent treatment with topical rebamipide eye drops four times a day for three months without any other topical agents to support wound healing of the ocular surface. Slit-lamp examination demonstrated a tiny pigmented lesion of the left eye (Fig. 2A) three months after the start of rebamipide treatment. After informed consent was obtained from the patient, the pigmented lesion was subsequently resected. Histologically, there was a small collection of nevus cells without cellular atypia. The noncancerous epithelium contained a large number of goblet cells (28 cells/HPF), where marked secretion of mucin-like substances from the goblet cells was noted (Fig. 2B). The surgical margin was free of nevus cells. As of January 2014, the patient was well without recurrence of the tumor. Informed consent was obtained from the patient prior to publication of the present study and the study was approved by the ethics committee of Hokkaido University.

## Discussion

Rebamipide has been used for patients with various tissue injuries in not only the ocular surface but also systemic mucosae such as the stomach. As one of its mechanisms of action, rebamipide is considered to induce goblet cells in the tissues in rabbit and mouse models (3,6). We recently demonstrated that rebamipide clearly influenced mucosal goblet cells in human conjunctival tissue (7), which verified *in vitro* evidence that rebamipide led to an elevation of the number of goblet cells in rat conjunctiva (4,5). The histological findings in the present case, furthermore, revealed that a 3-month administration of rebamipide resulted in not only in an increased number of goblet cells but also large quantities of mucin-like substances appearing on the ocular surface in a human patient. Thus, this phenomenon is clearly due to the effect of rebamipide and is not merely an epiphenomenon since the observed increase in goblet cell number is in agreement with findings obtained from animal experiments (3,6).

Clinical investigations have shown that topical rebamipide is useful for the treatment of patients with various corneal diseases, including dry eye (2,8), persistent corneal erosion (9), and lid wiper epitheliopathy (10). The present histological findings, therefore, may support the clinical usefulness of topical rebamipide in the ocular disorders described above. This study also indicates that rebamipide is useful for promoting favorable tissue repair in patients who have undergone surgery and/or biopsy in the ocular surface.
